# Human transcriptional interactome of chromatin contribute to gene co-expression

**DOI:** 10.1186/1471-2164-11-704

**Published:** 2010-12-14

**Authors:** Xiao Dong, Chao Li, Yunqin Chen, Guohui Ding, Yixue Li

**Affiliations:** 1 Key Lab of Systems Biology, Shanghai Institutes for Biological Sciences, Chinese Academy of Sciences, Shanghai, PR China; 2 Graduate School of Chinese Academy of Sciences, Beijing, PR China; 3 School of Life Science, University of Science and Technology of China, Hefei, PR China; 4 School of Life Sciences and Technology, Tongji University, Shanghai, PR China; 5 Shanghai Center for Bioinformation Technology, Shanghai, PR China

## Abstract

**Background:**

Transcriptional interactome of chromatin is one of the important mechanisms in gene transcription regulation. By chromatin conformation capture and 3D FISH experiments, several chromatin interactions cases among sequence-distant genes or even inter-chromatin genes were reported. However, on genomics level, there is still little evidence to support these mechanisms. Recently based on Hi-C experiment, a genome-wide picture of chromatin interactions in human cells was presented. It provides a useful material for analysing whether the mechanism of transcriptional interactome is common.

**Results:**

The main work here is to demonstrate whether the effects of transcriptional interactome on gene co-expression exist on genomic level. While controlling the effects of transcription factors control similarities (TCS), we tested the correlation between Hi-C interaction and the mutual ranks of gene co-expression rates (provided by COXPRESdb) of intra-chromatin gene pairs. We used 6,084 genes with both TF annotation and co-expression information, and matched them into 273,458 pairs with similar Hi-C interaction ranks in different cell types. The results illustrate that co-expression is strongly associated with chromatin interaction. Further analysis using GO annotation reveals potential correlation between gene function similarity, Hi-C interaction and their co-expression.

**Conclusions:**

According to the results in this research, the intra-chromatin interactome may have relation to gene function and associate with co-expression. This study provides evidence for illustrating the effect of transcriptional interactome on transcription regulation.

## Background

Gene transcription regulation is one of the important processes in biology. In eukaryotic cells, effect of highly compartmentalized nucleus on gene transcription regulation has come into notice. By experimental techniques, such as chromosome conformation capture [1] (3C) and interphase fluorescent *in situ *hybridization (FISH), the spatial associations between specific genes could be detected [2]. They provided accumulating data to study the of 'gene expression in 3D' [3].

Mechanisms such as transcription factory[4,5] and nucleus speckles[6-8], know as the "transcriptional interactome" were proposed, and spatial linkage of sequence-distant but function-related genes were revealed in case studies, although there are still debates on mechanisms [5,8]. Recently, the only "genome-wide study" was reported to demonstrate transcription interactions [5]. However it still focused on a small set of genes (mouse globin associated genes) rather than the whole genome. And still as J. Lawrence et al. once pointed out, 'a more challenging question for future studies is to determine whether the level of expression is indeed influenced by nuclear and chromosomal organization'[9].

Recently based on Hi-C experiment, a genome-wide picture of chromatin interaction in human gm06990 and K562 cells was reported [10]. As being demonstrated, Hi-C interactions can be applied as a measurement of spatial distance and chromatin organizations [10]. We used Hi-C interactions to evaluate whether on genomics level, spatial distances or chromatin structures have potential effect on transcription co-regulation. As the inter chromatin interactions are too small comparing to intra in Hi-C interactions [10], we focused only on intra-chromatin gene pairs.

## Results and Discussion

### Hi-C interaction correlates with co-expression in sequence-distant gene pairs when controlling transcription control similarity

To estimate the effect of chromatin organization, we test the correlation between Hi-C interaction (observed Hi-C interaction numbers, OH, and Pearson correlation coeffecient of them, PC, of 1 M and 100 k resolution of both human gm06990 and K562 cells, from Ref. [10], see Methods for details) and mutual ranks of gene co-expression rates (provided by COXPRESdb[11]). We controlled the effect of transcription factors on co-expression using transcription control similarity (TCS, see Methods for details) [12], and used 6,084 genes with both TF annotation (from ITFP[13]) and co-expression information for analyses. Then, 960,507 intra chromatin gene pairs were extracted by these genes. The Hi-C interactions between genes were represented by the interactions between the chromatin units where genes locate on. As gene co-expression rates are calculated among multiple tissues and cell types, 273,458 gene pairs with similar ranks of Hi-C interaction (ranks difference <5%) in the two cell types are finally used for analysis. We coined a term 'normalized distance' to measure the sequence distance between genes (see Methods for details).

**Figure 1:**
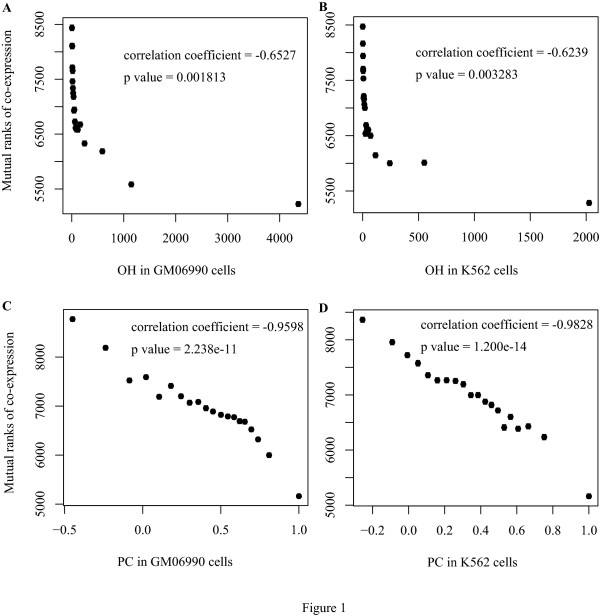
**Hi-C interactions correlate with mutual ranks of gene co-expression in intra chromosome gene pairs**. For all gene pairs within a chromatin, OH and PC of GM06990 and K562 cells are plotted against the mutual rank of gene co-expression. Error bars represent the standard error. For all figure panels A, B, C & D, p values lower than 0.0125 are significant, under Bonferroni corrections.

All pair-wise correlations between Hi-C interaction, ranks of gene co-expression, TCS and normalized distance were tested. As expected, the effect of TCS and normalized distance on co-expression could be observed (Additional File 1 & Additional File 2). Hi-C interactions strongly correlate with gene co-expression (Figure. 1), but potential association of TCS and normalized distance on Hi-C interaction exist (Additional File 3 & Additional File 4). It is difficult to explain the direct biological relationship between Hi-C interactions and gene co-expression.

**Figure 2:**
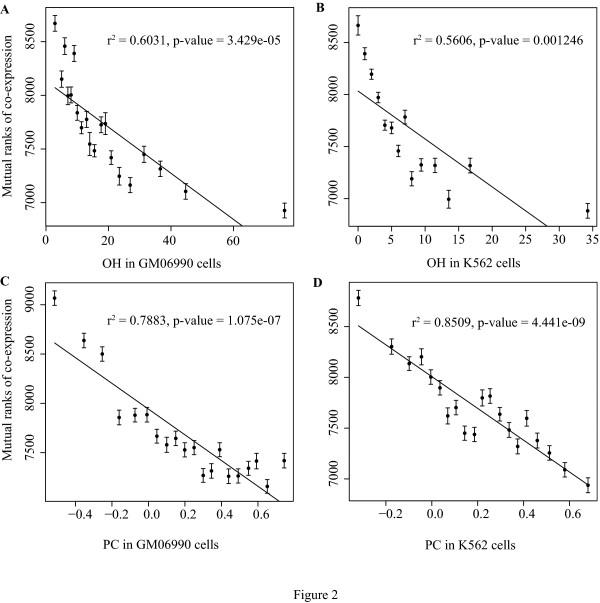
**Hi-C interactions correlate with mutual ranks of gene co-expression in sequence-distant gene pairs**. We took all the gene pairs within a same chromatin with normalised distance >0.2, and ploted their Hi-C interactions against mutual ranks of co-expression, and gene pairs with smaller ranks have more similar expression profiles. Error bars represent the standard error. For all figure panels A, B, C & D, p values lower than 0.0125 are significant, under Bonferroni corrections.

It was suggested that intra chromatin sequence-distant genes may be spatially close to each other and activated for expression[12]. Therefore, we focused on these pairs to test whether in common all spatially nearby intra-chromatin genes show co-expression. Noting that the correlation between normalized distance and co-expression is weak when normalized distance is bigger than 0.2 (Additional File 2). We chose was this point as the cut-off to select sequence-distant genes pairs for further analyses. For these selected pairs, the correlation between normalized distance and PC is not significant (*p *= 0.0606 and 0.0884, in GM06990 and K562 cells separately). And also, no significant correlation could be observed between TCS and the Hi-C interactions (for OH, *p *= 0.0546 and 0.1754, in GM06990 and K562 cells separately; for PC, *p *= 0.0208 and 0.1807). In such circumstances, there is an obvious trend that genes with more Hi-C interactions between them, would more likely to co-express (Figure. 2). It implies the potential genome-wide influence of chromatin struture on gene transcription regulation.

In most gene pairs, TCS equal to zero (107881 pairs out of 116834). Thus, we further divided them into two groups, equal or not to zero, which may provide us a better control for TCS. Correlation analyses were carried out as same as Figure. 1 and Figure. 2. In both groups, co-expressions are correlated with Hi-C interactions. (Additional File 5 & Additional File 6).

According to the above results, we show significant correlation between Hi-C interactoions and the mutual ranks of gene coexpression. However, the rank values we presented in those figures (Figure. 1, Figure. 2 and Additional File 1 to 6) are much higher than those that are used to construct gene co-expression networks [11]. In COXPRESdb, three levels of mutual ranks are used to construct networks (less than 5, 5 to 30, 30 to 50) [11]. We focused on 5735 co-expressed pairs which get mutual ranks ≤50, and found that they have much more numbers of interaction than other pairs (Additional File 7). Moreover, for co-expressed genes, there is a similar trend between the mutual ranks of gene co-expression and Hi-C interactions (Additional File 8).

### Function-related genes are close to each other and more likely to co-express

**Figure 3:**
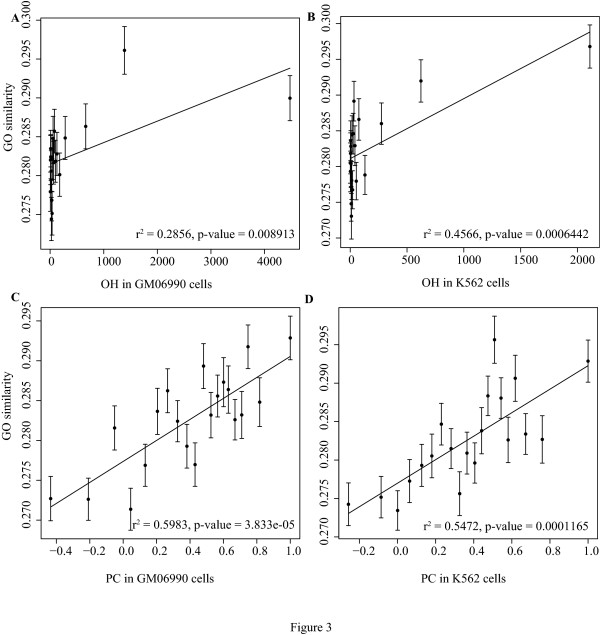
**Hi-C interactions correlate with gene function similarity**. All gene pairs within a same chromatin were divided into groups of equal size according to their Hi-C interactions, and we ploted their Hi-C interactions against the GO term similarity calculated by Wang's method[14]. Error bars represent the standard error. For all figure panels A, B, C & D, p values lower than 0.0125 are significant, under Bonferroni corrections.

We further asked whether intra-chromatin function-related genes are closer to each other as demonstrated by case studies before [5], and what their effects on co-expression are. GO term similarity [14,15] in biological process were used to represent functional relationship between genes. We found significant co-relation between GO similarity and Hi-C interaction for all the 273,458 gene pairs (Figure. 3). It suggests that function-related genes are close in space, though such correlations are weak in all intra chromatin pairs. The correlation between GO similarity and co-expression is nearly the same as between Hi-C interaction and co-expression (Figure. 3 and Additional File 9). This suggests that genes with relevant functions, would be more likely close to each other, and such phenomenon would affect transcription regulation.

### Transcription factory and open/closed chromatin may be the underlying mechanism

Why co-localized genes turn to have a similar kind of expression profile? It was suggested that transcription active sites exist in nucleus, such as Pol II-enriched transcription factories [4] and splicing-factor-enriched speckles [7]. Genes locate on such sites are more actively transcribed, and could move in and out to be transcriptionally active or quiescent [4,16]. Such movement may be an important factor in gene transcription regulation [2,4,17]. In Hi-C experiment, formaldehyde is used to cross-link cells, resulting in covalent links between spatially adjacent chromatin segments [10]. This procedure is just the same as 3C[1] and 4C[18], which are used to support the transcription factory mechanism[3], and implies the dynamic instinct of gene localization. So our result based on Hi-C could be good evidence to illustrate that transcription factories are genome-wide common in human nucleus.

Besides the transcription active sites in nucleus, chromatin modification or structure also plays a significant role in gene transcription regulation [12,19]. One of the well-known models for such structure is open and closed chromatin [10,12]. Under an assumption that sequence-neighboring genes have a similar chromatin structure, N. Batada et al. found that these genes have higher co-expression rates than separate ones [12]. They also suggested that the transformation of chromatin structure between open and closed state - chromatin remodelling, is a major source of co-expression of linked genes[12]. In the Hi-C interactions, PC is an indicator of these structure [10]. So our results could provide common evidence that open and closed chromatin structures the regulate gene expression without N. Batada's assumption[12].

## Conclusions

We have provided genome wide evidence of the correlation between Hi-C interaction and co-expression of sequence-distant TF-annotated intra-chromatin gene pairs. Our results highlight a possible general and independent effect of transcription interactome, on gene transcription regulation, and such effect may be gene-function related. However, it should be noticed that there are still some difficulties to get a definite conclusion. First, Lieberman-Aiden's study is still the only human Hi-C data available on just two cell lines [10]. It is hard to distinguish the dynamic and stable chromatin interactions among different cell types. Second, the Hi-C data has a low resolution, that chromatins are divided into 1 M and 100 k units, and the average interactions between units are counted. One unit may include many genes. Therefore, statistics based upon it, is not prescise. We hope in the future, when more is available, the chromatin-level of gene transcription regulation would be more precisely demonstrated.

## Methods

The Hi-C interaction data from Lieberman-Aiden's article can be accessed through the GEO data base with an accession no. GSE18199 [10]. Both observed interactions (OH) and Pearson correlation of them (PC) were used in our analyses. It was pointed out that OH can be applied as a measurement of spatial distance and PC is an indicator of chromtin structure [10]. In GEO, PC of X chromosome of K562 cell is missing, so we calculated it from the OH using the original method [10]. Mutual ranks of gene coexpression scores are from COXPRESdb [11], which includes co-expression data for 19,777 genes in human. The information about human transcription factor are from the Integrated Transcription Factor Platform (ITFP, version 1.0 Aug 2008)[13], which under current release includes 4,105 putative TFs and 69,496 potential TF-target pairs for human. And for the GO anotation of human genes, we use the GO.db package (version 2.4.1) for R (http://www.r-project.org/).

Normalized distance is defined as the sequence distance (bp) between two intra-chromasome genes, over the total length (bp) of their chromosome. To measure transcription control similarity (TCS), we use Batada's defination [12]. They define that transcription control similarity for a given gene pair "as one minus the number of transcription factors that bind one but not both the genes, divided by the sum of the number of regulator-target interactions." [12].

We took 6,084 genes within both COXPRESdb [11] and ITFP [13] for analyses. There are 960,507 intra chromatin gene pairs from them. The Hi-C interaction of gene pairs is calculated by the weighted average of interactions of 1 M chromatin units where genes locate on. We calculated Hi-C interaction rank difference of gene pairs of the two cell types, and choose 273,458 pairs with rank difference <5% for further analyses.

For the 273,458 pairs, we tested pair-wise correlation between Hi-C interaction, mutual ranks of gene co-expression rate, TCS and normalized distance. Then we choose 116,834 pairs with normalized distance ≥0.2, and again tested the above correlations. And we further divide the pairs into two groups according to their TCS values, equal or not to zero, to test the correlations. For all correlation analysis (all figures and correlation test in the article), we divided all samples into 20 groups according to their horizontal axis values. The number of samples in each group is expected to be similar. However, we find that in several figures some groups are missing because there are too many samples with a same x-axis value. (In Figure. 2B, group 3, 5, 7, 9, 11 & 14 are missing. In Additional File 5B, group 3, 5, 7, 9, 11 & 14 are missing. In Additional File 6B, group 3, 5, 7, 9 & 12 are missing.) Group's average values for both horizontal and vertical values are calculated for analysis. Because gene pairs are significantly not equally distributed according to the x-axis values, so we do not divide them by using the same size of interval of x-axe as many studies did.

For the gene function similarity analysis, we used Wang's method for measuring the semantic similarity of GO terms[14], and used an R package, GOSemSim[15] to achieve it.

## Authors' contributions

Both Xiao Dong and Chao Li carried out all the analysis in this study, Xiao Dong conceived of the study. Yixue Li and Guohui Ding participated in the study design and coordination, and helped to draft the manuscript. All authors read and approved the final manuscript.

## Supplementary Material

Additional file 1 **Transcription control similarity (TCS) has a strong influence on gene co-expression as expected**. For all gene pairs within a chromatin, transcription control similarity, as expected has a strong influence on the ranks of gene co-expression rates. Error bars represent standard error. Click here for file

Additional file 2 **Neighboring genes are more likely to be co-expressed**. As being pointed out, neighboring genes are more likely to be co-expressed. Error bars represent standard error. Click here for file

Additional file 3 **For all gene pairs, association between TCS and Hi-C interactions exist**. For all gene pairs, we plotted their Hi-C interactions against their TCS. And those with higher interactions, their TCS are more similar. Error bars represent standard error. Click here for file

Additional file 4 **Normalized distance has a significant effect on Hi-C interactions as expected**. As expected, for all gene pairs the HI-C interactions of neighboring genes are significantly stronger than those distant ones. Error bars represent standard error. Click here for file

Additional file 5 **Correlation between Hi-C interaction and gene co-expression for gene pairs, which TCS are zero**. For the pairs that TCS are zero, Hi-C interactions are plotted against the ranks of gene co-expression rates. Genes with more Hi-C interactions between them, would more likely to co-express. Error bars represent standard error. For all figure panels A, B, C & D, p values lower than 0.0125 are significant, under Bonferroni corrections. Click here for file

Additional file 6 **Correlation between Hi-C interaction and gene co-expression for gene pairs, which TCS are not zero**. For the pairs that TCS are not zero, Hi-C interactions are plotted against the ranks of gene co-expression rates. Genes with more Hi-C interactions between them, would more likely to co-express. Error bars represent standard error. For all figure panels A, B, C & D, p values lower than 0.0125 are significant, under Bonferroni corrections. Click here for file

Additional file 7 **Co-expressed gene pairs have more Hi-C interactions than all pairs**. T tests are used to test the difference between Hi-C interactions of co-expressed gene pairs and all pairs. In co-expressed gene pairs, the interactions are significantly higher. P values lower than 0.0125 are significant, under Bonferroni corrections. Click here for file

Additional file 8 **Correlations between co-expression and Hi-C interaction in co-expressed gene pairs**. For co-expressed gene pairs (mutual ranks less than or equal to 50), we could also observed the correlation between Hi-C interaction and their mutual ranks of co-expression. For all figure panels A, B, C & D, p values lower than 0.0125 are significant, under Bonferroni corrections. Click here for file

Additional file 9 **Gene's GO similarities are related with their co-expression **We plotted GO similarities between genes against their ranks of co-expression rates. Error bars represent standard error. Click here for file
