# Supplementary figures and images for: Human transcriptional interactome of chromatin contribute to gene co-expression
Source: BMC Genomics. 2010 Dec 14;11:704. doi: 10.1186/1471-2164-11-704 (PMC3053592; doi:10.1186/1471-2164-11-704)

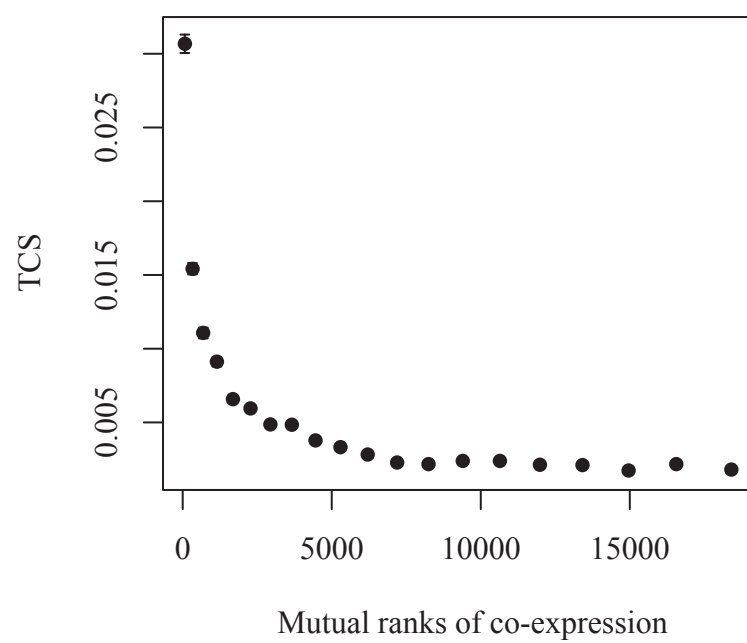

Supplemental Figure 1

Supplement: Additional file 1 — Transcription control similarity (TCS) has a strong influence on gene co-expression as expected. For all gene pairs within a chromatin, transcription control similarity, as expected has a strong influence on the ranks of gene co-expression rates. Error bars represent standard error. [file 1471-2164-11-704-S1.PDF]

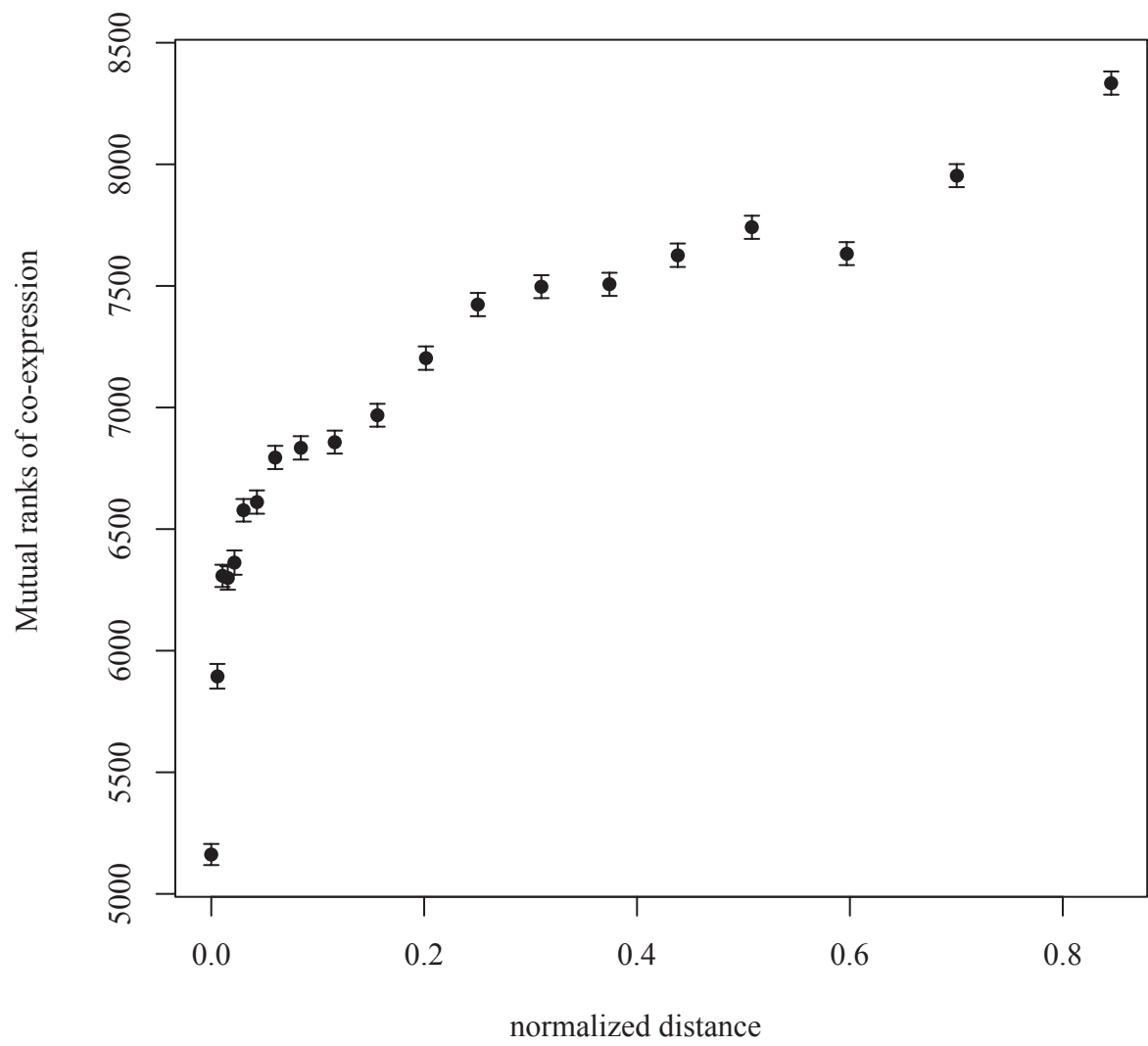

Supplemental Figure 2

Supplement: Additional file 2 — Neighboring genes are more likely to be co-expressed. As being pointed out, neighboring genes are more likely to be co-expressed. Error bars represent standard error. [file 1471-2164-11-704-S2.PDF]

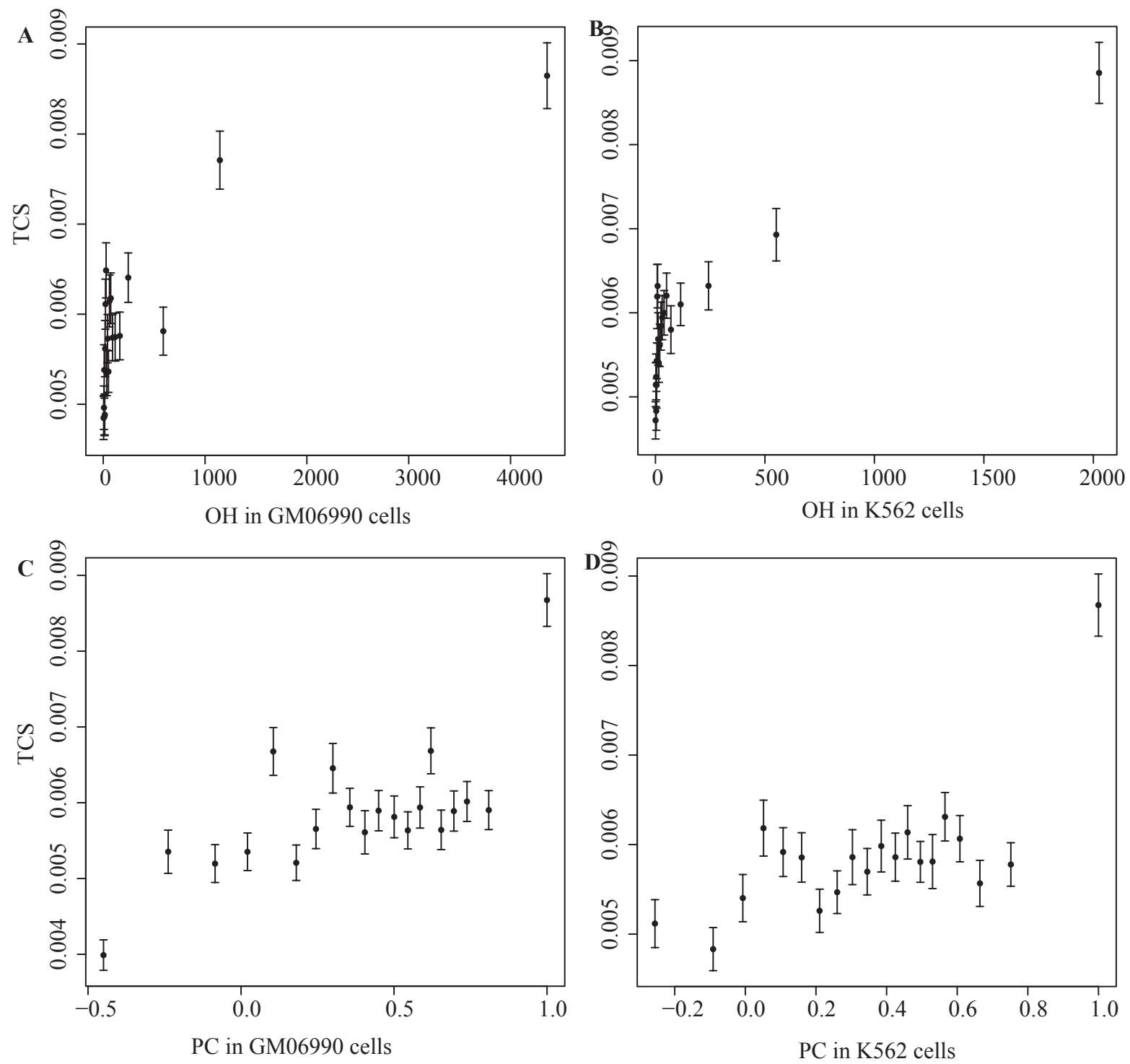

Supplemental Figure 3

Supplement: Additional file 3 — For all gene pairs, association between TCS and Hi-C interactions exist. For all gene pairs, we plotted their Hi-C interactions against their TCS. And those with higher interactions, their TCS are more similar. Error bars represent standard error. [file 1471-2164-11-704-S3.PDF]

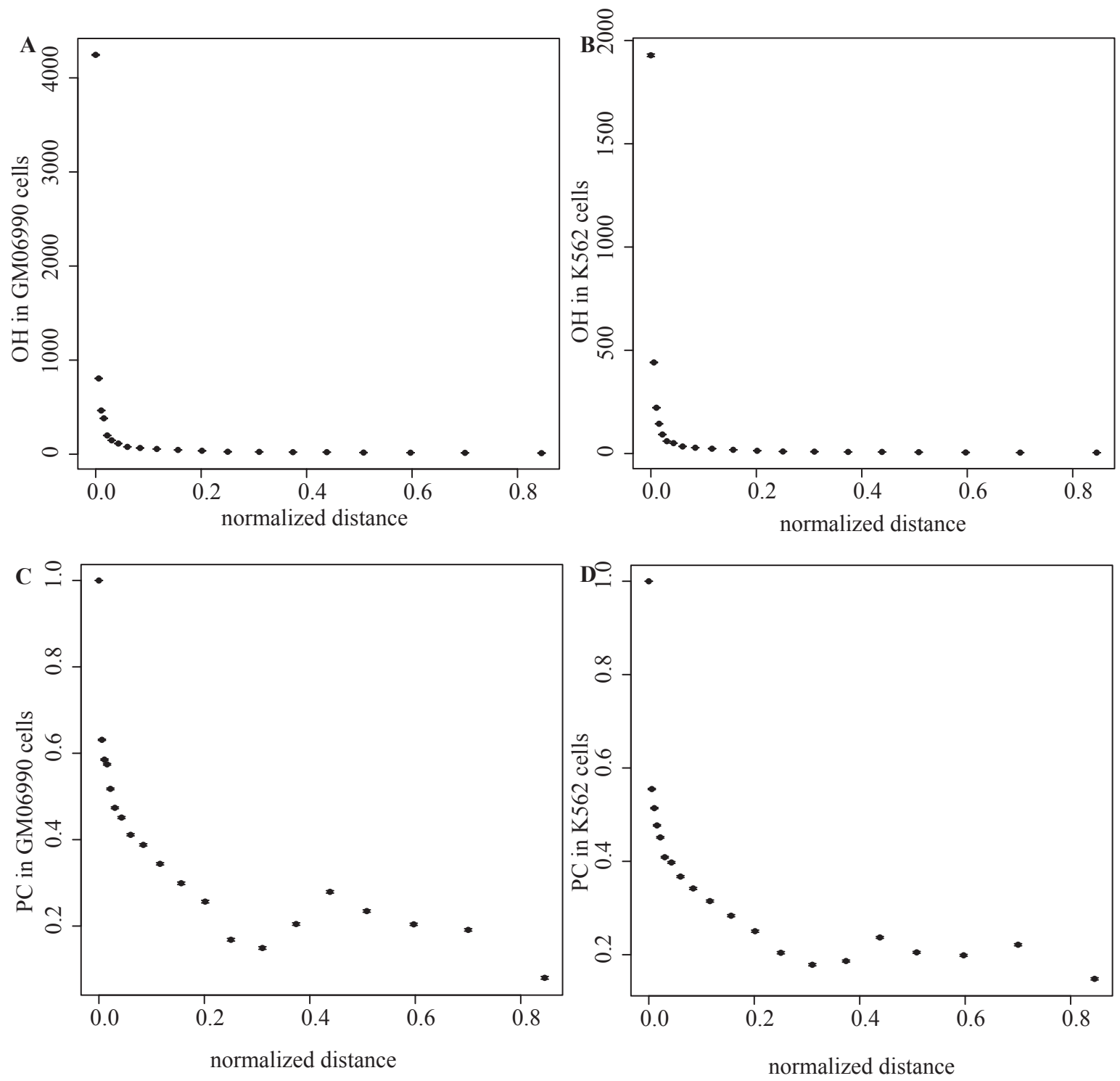

Supplemental Figure 4

Supplement: Additional file 4 — Normalized distance has a significant effect on Hi-C interactions as expected. As expected, for all gene pairs the HI-C interactions of neighboring genes are significantly stronger than those distant ones. Error bars represent standard error. [file 1471-2164-11-704-S4.PDF]

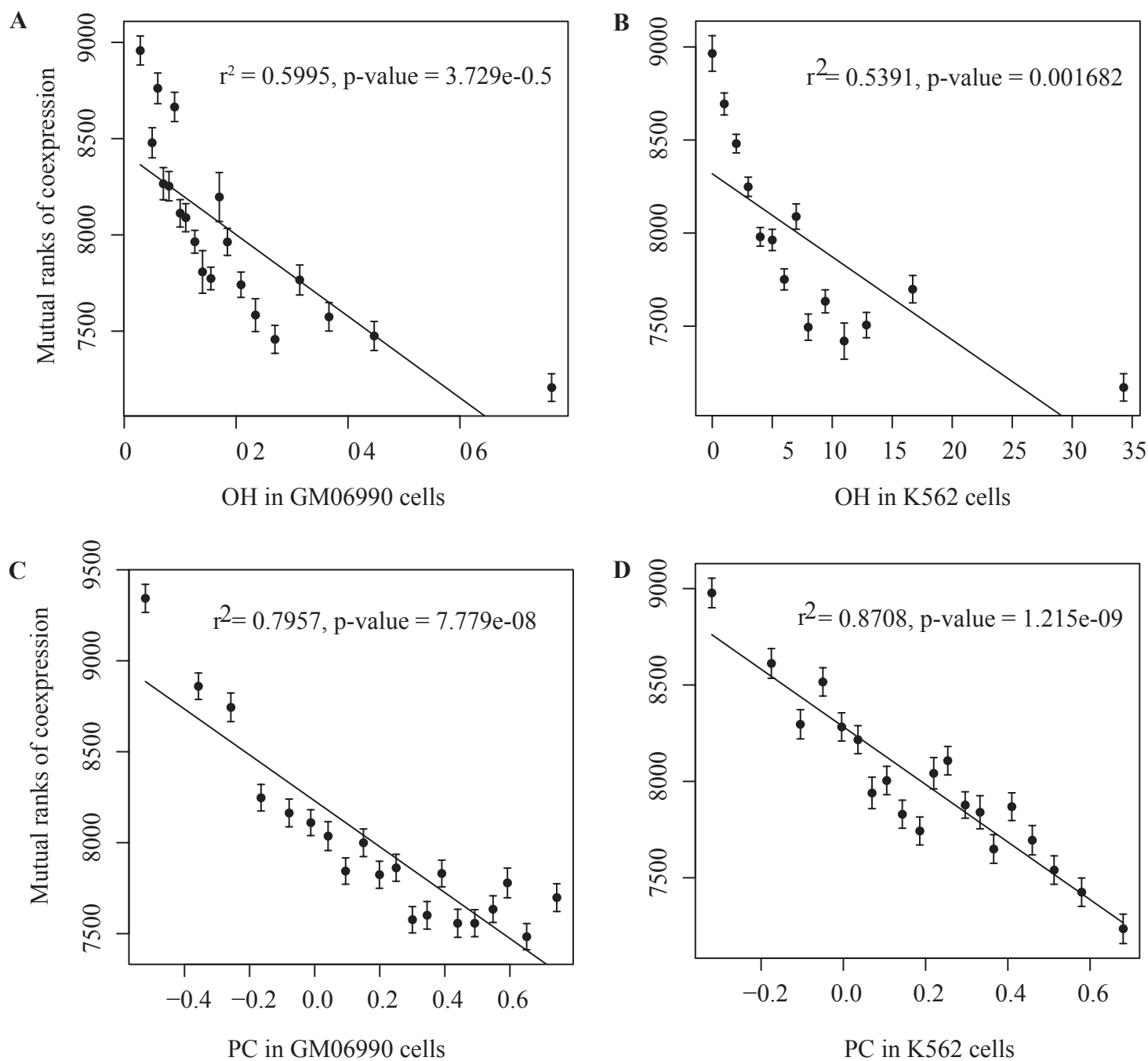

Supplemental Figure 5

Supplement: Additional file 5 — Correlation between Hi-C interaction and gene co-expression for gene pairs, which TCS are zero. For the pairs that TCS are zero, Hi-C interactions are plotted against the ranks of gene co-expression rates. Genes with more Hi-C interactions between them, would more likely to co-express. Error bars represent standard error. For all figure panels A, B, C & D, p values lower than 0.0125 are significant, under Bonferroni corrections. [file 1471-2164-11-704-S5.PDF]

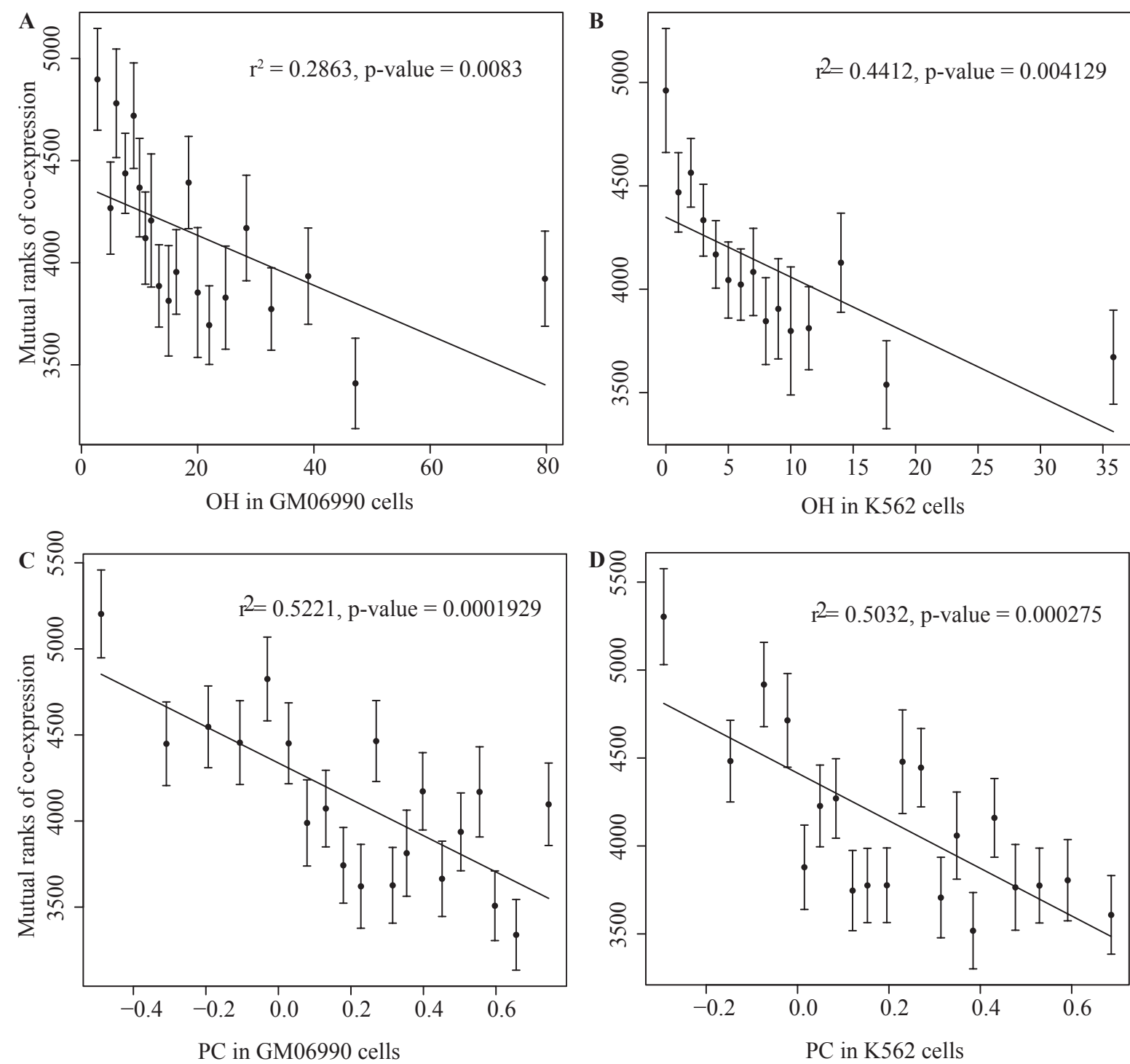

Supplemental Figure 6

Supplement: Additional file 6 — Correlation between Hi-C interaction and gene co-expression for gene pairs, which TCS are not zero. For the pairs that TCS are not zero, Hi-C interactions are plotted against the ranks of gene co-expression rates. Genes with more Hi-C interactions between them, would more likely to co-express. Error bars represent standard error. For all figure panels A, B, C & D, p values lower than 0.0125 are significant, under Bonferroni corrections. [file 1471-2164-11-704-S6.PDF]

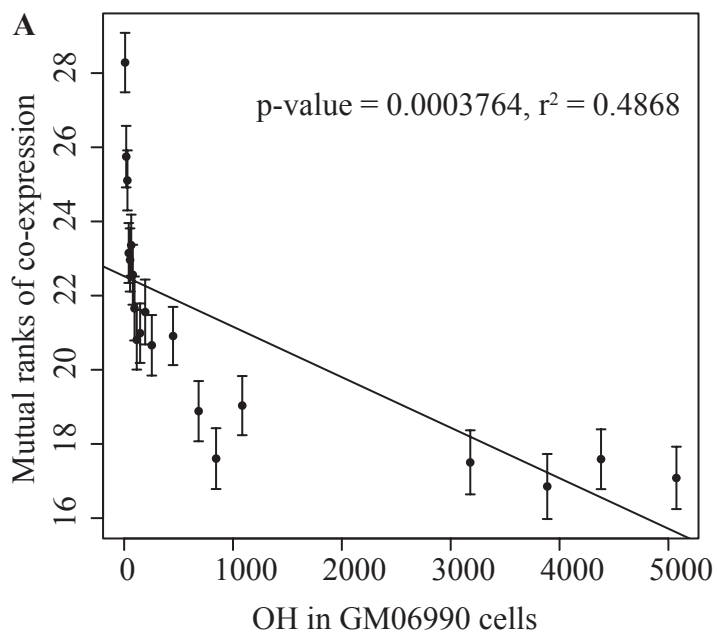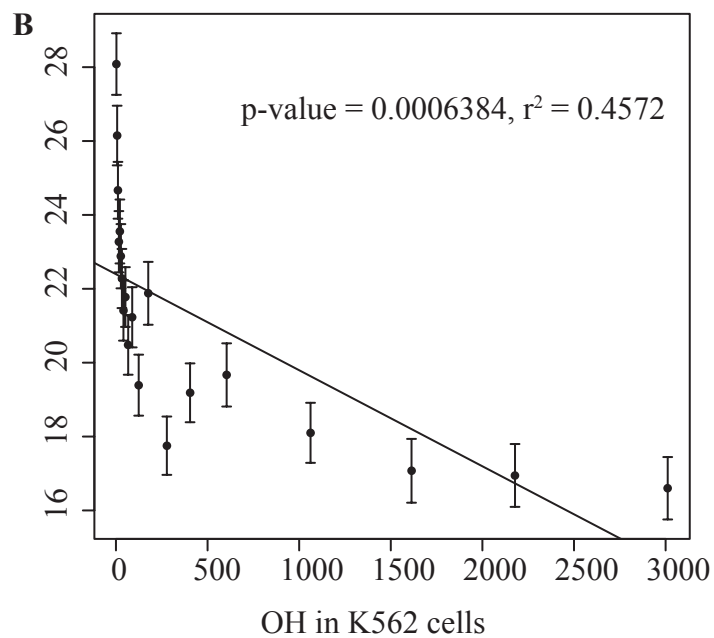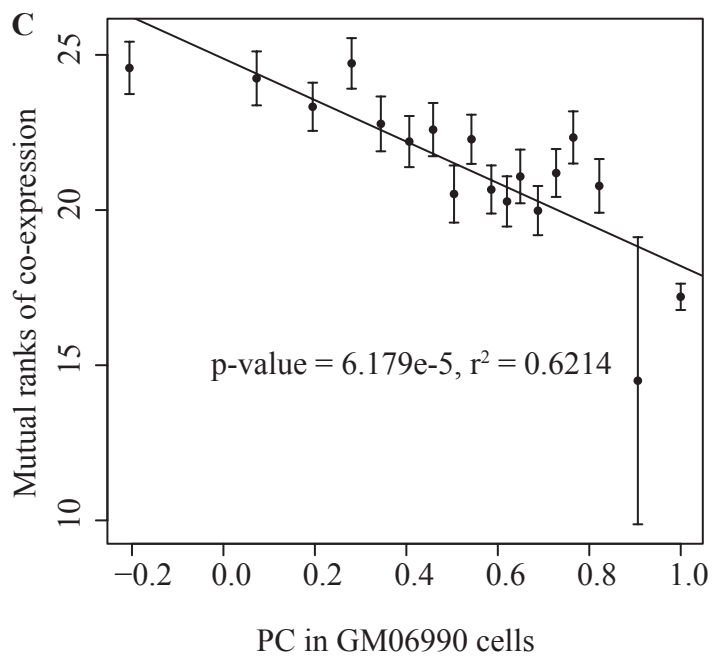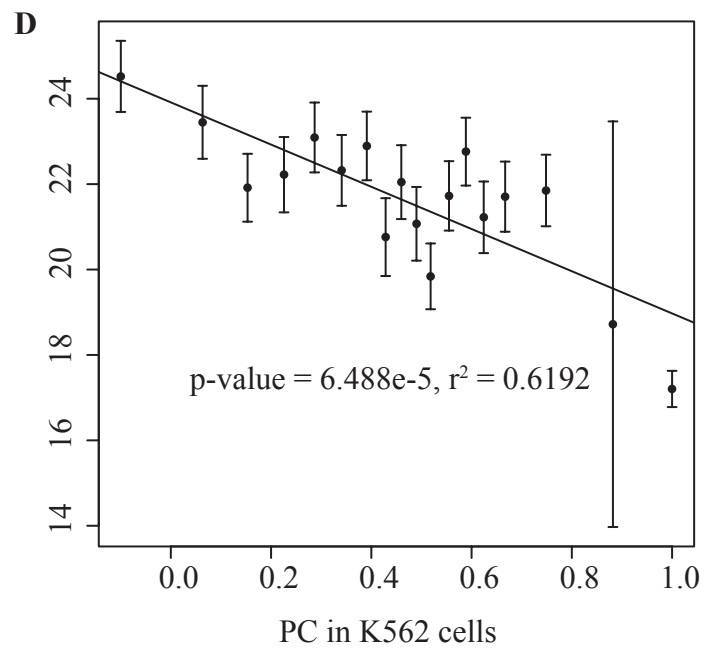

Supplemental Figure 7

Supplement: Additional file 8 — Correlations between co-expression and Hi-C interaction in co-expressed gene pairs. For co-expressed gene pairs (mutual ranks less than or equal to 50), we could also observed the correlation between Hi-C interaction and their mutual ranks of co-expression. For all figure panels A, B, C & D, p values lower than 0.0125 are significant, under Bonferroni corrections. [file 1471-2164-11-704-S8.PDF]

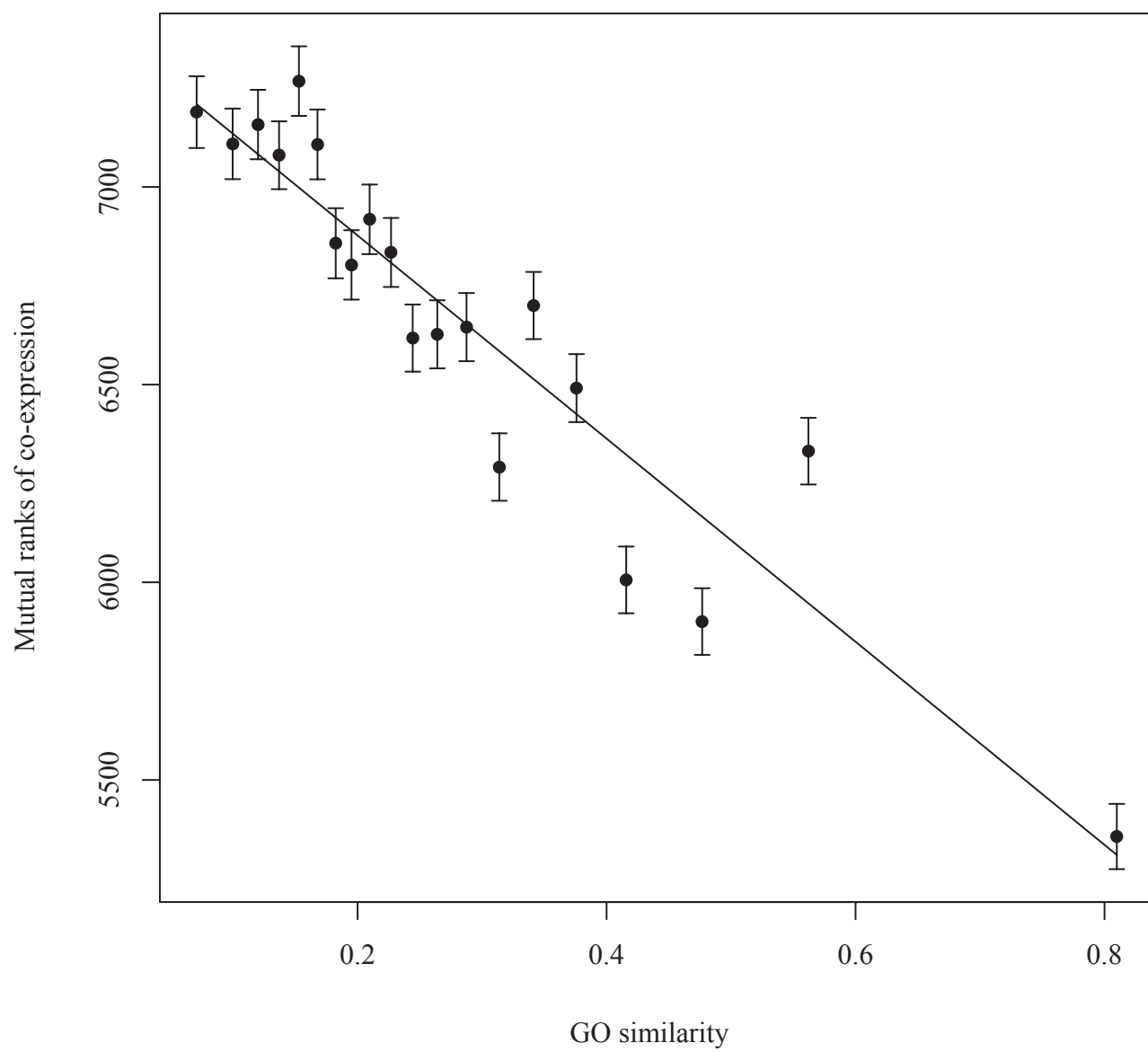

Supplemental Figure 8

Supplement: Additional file 9 — Gene's GO similarities are related with their co-expression We plotted GO similarities between genes against their ranks of co-expression rates. Error bars represent standard error. [file 1471-2164-11-704-S9.PDF]
